# The ASHA (Hope) Project: Testing an Integrated Depression Treatment and Economic Strengthening Intervention in Rural Bangladesh: A Pilot Randomized Controlled Trial

**DOI:** 10.3390/ijerph18010279

**Published:** 2021-01-01

**Authors:** Alison Karasz, Shabnam Anne, Jena Derakhshani Hamadani, Fahmida Tofail

**Affiliations:** 1 Department of Family and Social Medicine, Albert Einstein College of Medicine, 1300 Morris Park Avenue, Bronx, NY 10461, USA; alison.karasz@einsteinmed.org; 2 Nutrition and Clinical Services Division, International Centre for Diarrhoeal Disease Research, Bangladesh (ICDDR, B), Mohakhali, Dhaka 1212, Bangladesh; anne.shabnam230@gmail.com; 3 Maternal and Child Health Division, International Centre for Diarrhoeal Disease Research, Bangladesh (ICDDR, B), Mohakhali, Dhaka 1212, Bangladesh; jena@icddrb.org

**Keywords:** depression, poverty, ASHA, PHQ-9, asset, cash-transfer, tension, self-esteem, matched savings, Bangladesh, structural interventions

## Abstract

Depression, a debilitating disorder, is highly prevalent among low-income women in low- and middle-income countries. Standard psychotherapeutic approaches may be helpful, but low treatment uptake, low retention, and transient treatment effects reduce the benefit of therapy. This pilot randomized controlled trial examined the effectiveness and feasibility of an integrated depression treatment/economic strengthening intervention. The study took place in two villages in the Sirajganj district in rural Bangladesh. Forty-eight low-income women with depressive symptoms (Patient Health Questionnaire (PHQ-9) score ≥ 10) were recruited and randomized to intervention or control arms. The intervention included a six-month group-based, fortnightly depression management and financial literacy intervention, which was followed by a cash-transfer of $186 (equivalent to the cost of two goats) at 12 months’ follow-up. The cash transfer could be used to purchase a productive asset (e.g., agricultural animals). The control arm received no intervention. Findings showed significant reduction in depression scores in the intervention group. The mean PHQ-9 score decreased from 14.5 to 5.5 (B ± SE, −9.2 ± 0.8 95% CI −10.9, −7.5, *p* < 0.01) compared to no change in the control group. Most other psycho-social outcomes, including tension, self-esteem, hope, social-support, and participation in household economic decision-making, also improved with intervention. An integrated depression treatment and financial empowerment intervention was found to be highly effective among rural low-income women with depression. Next steps involve formal testing of the model in a larger trial.

## 1. Introduction

Depression is a frequent consequence of poverty and its sequelae, including hunger, deprivation, social marginalization, and hopelessness [1,2,3,4,5,6]. At the same time, depression contributes to poverty and financial hardship via reduced economic productivity and impaired decision making [7,8,9]. Together, poverty and depression link with other health conditions—e.g., HIV, diabetes, and child malnutrition [10,11]—to form intractable global syndemics that defy easy solutions. Creative, innovative approaches that effectively interrupt the cycle of poverty, depression, and disease are necessary.

Depression is a global gender disparity, with prevalence rates nearly twice as high in women compared to men [12,13]. However, the excess burden of depression born by women in South Asian societies may exceed that of other parts of the world [14,15]. Traditional South Asian gender roles and practices often devalue women, reduce their share of household resources, and limit access to education, employment, and opportunities to build social capital [16,17,18,19,20,21]. In addition to the burden of psychological suffering, women’s depression has important consequences for children’s health. Among low-income mothers in low- and middle-income countries, depression has been linked to poor social, health, and cognitive outcomes in children, especially malnutrition, stunting, and cognitive delays [22,23].

The global mental health (GMH) movement, promoted in recent years by researchers and policy makers at the WHO [24,25], seeks to address the pandemic of depression in low- and middle-income countries (LMIC) lacking a developed mental health workforce. In this approach, therapies developed in the west—cognitive behavioral, problem solving, and interpersonal therapies—are delivered via nonprofessional community health workers—an approach known as “task sharing”. A recent meta-analysis found that task sharing interventions generally outperform no-treatment/treatment-as-usual controls [26], although recent large-scale studies in South Asia have shown disappointing results [27,28].

Task-sharing therapeutic strategies, thus, hold promise. Yet, the GMH movement is not without its critics. One concern is that the movement shifts the emphasis from the major causes of the global depression epidemic—poverty, violence, and inequality—to a medical model of depression as a problem of individual pathology, in keeping with individualistic philosophies of Western societies [29,30]. Advocates of the GMH movement have noted that psychotherapy treatment works—at least in theory—by empowering individuals to make changes in their lives. Yet, in many settings, particularly in LMIC, individuals lack autonomy and resources to make such changes [31].

Furthermore, the large, decades-old literature on depression treatment outcomes in the West suggests a note of caution regarding the benefits of depression treatment in deprived settings. Income itself is a powerful moderator of treatment outcomes in depression. Large-scale depression treatment studies in the US have found that lower income is associated with weaker treatment effects [32,33,34,35] and is a stronger indicator of treatment response than clinical variables [36].

Second, mental health treatment effects are transient [37]. A recent review of the long-term efficacy of psychotherapy (44 studies) found that intervention–control differences declined rapidly starting at about six months’ post-baseline. In evaluations of depression treatment in LMIC, study design—specifically, the length of the follow-up period—influences outcomes, with short-term assessments tending to show greater benefits [38,39,40].

Finally, poverty reduces treatment uptake and engagement—even when depression treatment is available. Evidence suggests that people in low-income communities endorse conceptual models of depression that emphasize the social and economic causes of suffering. They are pessimistic about the benefits of standard depression treatments, whether pharmacological or psychotherapeutic [21,41,42,43,44,45,46]. Consequently, low-income people, compared with the more affluent, are less likely to accept or adhere to conventional depression treatments [47,48,49].

With the goal of developing a new treatment model for low-resource settings that would a) engage and retain low-income individuals and b) have potential to create lasting benefits, we developed an integrated depression treatment/economic strengthening intervention using matched savings accounts (MSA). Research shows that MSAs, with other types of cash transfer programs, can increase hope, confidence, self-esteem, and future orientation, as well as mental health and wellbeing [39,50,51,52,53]. Among low-income women, asset accumulation can improve social status within the community and bargaining power within the household [53,54].

ASHA (“hope” in Bengali), a 12-session, group-based treatment delivered by peer health workers, was developed through a research–community partnership among researchers, clinicians, activists, and South Asian women immigrants in New York City [55]. In the ASHA intervention, participants receive financial literacy education, matched savings accounts, and business training, followed by an asset transfer. The funds may be used for purchases that enhance economic independence (e.g., training, education, small business capitalization). The model was piloted in a group of low-income Bangladeshi women in the US and showed promise in reducing depression and retaining participants in treatment [55]. This paper reports on a pilot test of the ASHA model in an economically deprived area of rural Bangladesh. We tested whether the ASHA intervention reduced depressive symptoms at 12 months compared to a no-treatment control.

## 2. Materials and Methods

### 2.1. Study Sites

Two villages in the Sirajganj district were selected as study sites. This flood-prone district, situated by the side of the river Jamuna, extends over a 2402.05 sq. km area. It is located 101 km from Dhaka, the capital of Bangladesh. Main income sources include agriculture, fishery, poultry, hatchery, and weaving. Average literacy is relatively low (male 51%, female 44%), and 53% of the population is landless. The villages are among those served by the National Development Programme (NDP), a national, non-governmental, non-profit organization in Sirajganj that provides services to villages in the region. The NDP was our community partner in this project. NDP health workers delivered the intervention and provided bank accounts and training on animal husbandry.

### 2.2. The Intervention

The intervention was delivered via a woman-centered framework emphasizing a woman’s right to respect, dignity, and care. Groups of twelve women met for two hours every other week for six months. The 12-session intervention included eight sessions of depression treatment and four sessions of financial literacy education, followed by a cash transfer. Following the 12-session intervention, women continued meeting with NDP local agricultural officers until they were ready to make their agricultural asset purchases.

The eight-session depression treatment protocol included (1) basic mental health literacy—understanding and identifying depression; (2) reducing negative cognitions; (3) improving interpersonal relationships; and (4) behavioral activation (increasing activity, engaging in pleasurable activities).

Financial literacy training was provided by the NDP and included sessions on savings, credit, and animal husbandry. Each woman opened a bank account at the beginning of the study and made regular deposits of approximately $2.5 (range $1.25–6.25) per month, which is equal to the cost of approximately 4 kg of rice in. Participants in the intervention were given the option of engaging in income-producing activities, such as tree planting, to earn small amounts of cash to make deposits. During the intervention period, the participants worked with NDP agricultural officers to plan for their asset purchase.

Each intervention group member was assigned a bandhobi (“friend”) partner within the group, with whom she was requested to make at least one contact between each group meeting. Group facilitators and other project staff assigned bandhobi pairs based on factors such as age and proximity. The goal of the bandhobi component was to increase social support and enhance therapeutic benefit. Participants in the intervention group received training in supportive listening and were expected to provide emotional as well as instrumental support to their bandhobis when appropriate.

At the 12-month point, participants were given an up to 6x match of their savings, with a maximum total of $186 (equivalent to the cost of two goats). Over 95% of participants chose to purchase an agricultural animal.

#### Training and Supervision of Peer Interventionists

Two part-time female facilitators from each center were recruited in each village. They received five-day manualized training, focused on program contents, informal counseling, and group facilitation skills. Participatory (learner-centered) approaches were used in the training. Techniques included encouraging discussion/avoiding lecture, active listening, using questions, sharing personal experiences where appropriate, encouraging learners to teach each other, valuing the perspective of each learner, and embracing humility [56].

### 2.3. Screening and Recruitment

**Figure 1:**
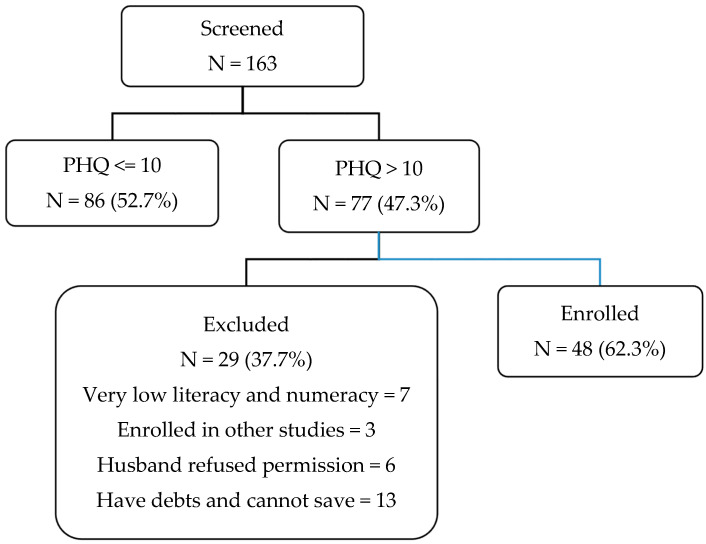
Enrollment.

Over a two-month period, peer workers visited homes in the two villages and screened 163 housewives. Inclusion criteria included the following: housewife; age between 18 and 40 years; depressive symptoms (Patient Health Questionnaire (PHQ-9) score ≥ 10); minimum literacy (participants were asked to read a short paragraph and write a sentence); ability to save a minimum of 50 takas (around $0.60/week). All participants agreed to be screened and consented. A convenience sample of 48 participants meeting eligibility criteria were enrolled in the study (see Figure 1). In this parallel trial, participants were randomized 1:1 through a computer-generated list of random numbers into the intervention (*n* = 24) or control arm (*n* = 24). After participating in the informed consent process, each woman underwent baseline assessment (T1), which was performed in her home, courtyard, or at the NDP center, depending on her preference. A trained master’s level psychologist conducted all assessments. Following the baseline interview, participants were randomized 1:1 to the intervention or control groups. The intervention group received the intervention; the control group received no treatment. The second interview (T2) was conducted 12 months’ post-baseline, following asset purchase. Participants received an honorarium of approximately $1.86 for participating in each interview.

### 2.4. Measures

#### 2.4.1. Depression 

The PHQ-9 is extensively used and validated in cross-cultural settings, and it was the main outcome measure for the study [57]. It consists of nine items, each with 4-point Likert responses (score range from 0 to 27: a higher score indicates more severe depression). It has been used frequently and has demonstrated reliability and validity in studies in Bangladesh [58,59]. A cut-off point of 10, indicating clinical depression, has been suggested [60]. 

#### 2.4.2. Other Measures 

*Cultural expressions of distress.* The Tension Scale includes psychological and somatic symptoms expressive of distress in the South Asian cultural context. It has been validated in a group of Bangladeshi women living in the United States [61]. A higher score indicates greater distress. *Social support* was measured using the Medical Outcomes Study (MOS) Social Support Survey [62]. A higher score indicates a greater level of support. *Household empowerment* was measured using the Mason Empowerment Scale, which has been widely used in development contexts [63]. It includes five subscales measuring empowerment domains: household economic decision making (a higher score indicating a greater level of wife’s participation in decision making about large and small purchases); decisions about family size (a higher score indicates a wife’s greater level of participation); wife’s relative geographic mobility vs. confinement to the home (a lower score indicates less confinement); exposure to physical violence or coercion (a lower score indicates lower exposure to violence or coercion); and gender role norms (a lower score indicates more egalitarian norms) [63]. *Hope/future orientation* was measured using the Trait Hope Scale [64] containing 12 questions with a 4-point Likert response; a higher score indicates greater hopefulness. *Self-esteem* was measured using the eight questions of Rosenberg Self-esteem scale [65]; a higher score indicates greater self-esteem. All instruments were pretested before use in the field.

### 2.5. Ethics Approval and Consent to Participate

The study was approved by the Institutional Review Board at Albert Einstein College of Medicine.

### 2.6. Data Analysis

Means and standard deviations for all variables were calculated. Using an intent-to-treat approach, we conducted independent-sample t tests for continuous variables and chi-squared tests for categorical variables. To control for baseline differences in a final step, we conducted a multiple linear regression analysis that included demographic variables. We entered and analyzed data using SPSS for Windows (version 17.0; SPSS Inc., Chicago, IL, USA), and *p*-values < 0.05 were considered statically significant.

## 3. Results

### 3.1. The Sample

For a description of the sample, see Table 1. Baseline data were collected in July 2013. The mean age of participants was 26.1 (SD = 4.6). Education varied widely across the sample, with a median of 5 (range 1–10) years of schooling. Most participants lived in extended family settings. Debt carried by households was reported by 38% of families and ranged from TK 500 to 15,000 ($6–185).

At baseline, the mean (SD) PHQ-9 score (not shown) was 14.5 (3.1), suggesting a moderate level of depression. Depression was slightly higher in Village 2, situated in a more flood-prone area than Village 1, although the difference was not significant (14.8 vs. 14.2, *p* = 0.45).

### 3.2. Outcomes

Table 2 presents the unadjusted psychosocial and clinical outcomes at 12 months’ post-baseline, reporting means and standard deviations of all the outcome variables before and after the intervention. Compared to controls, the intervention group reported a steep decline in depressive symptoms as measured by the PHQ-9. Mean symptoms decreased from 14.5 at baseline to 5.5 at 12 months in the intervention group, while no significant change was observed in the control group. Significant improvements were noted for other psychosocial variables, including the Tension Scale, Social Support Scale, and other psychosocial outcomes. Analyses of the Empowerment Scale found mixed results. At follow up, intervention participants reported significantly larger improvements in the degree of participation in financial decision making and greater reduction in experiences of physical or mental coercion than the non-intervention arm. However, participation in decision making regarding family size, geographic mobility, and gender role norms did not differ significantly across groups (Table 2). Controlling for baseline scores of education and age in multivariable-adjusted linear regression analysis (Table 3) did not alter the results or the calculated effect sizes.

In summary, the intervention group differed significantly on most outcomes in the expected direction. While our sample size was too small for mediational analyses, the intervention–control differences on most social and psychological measures indicated that such variables may mediate the impact of the intervention. Improvements in social support and household empowerment, for example, may account for some of the improvements in depressive symptoms.

### 3.3. Other Outcomes

Other program outcomes: *Retention*. There were no other withdrawals from the intervention or the research study. *Attendance*. Attendance was excellent: 100% of participants attended at least 10 sessions: the median number of sessions attended was 12. *Asset purchases.* All 24 participants in the intervention arm made their required deposits and qualified for a match of $186. Twenty-two of 24 women chose to use their assets to purchase an agricultural animal; one woman purchased equipment to make thread, and one rented a plot of land for vegetable cultivation. *Bandhobi program outcomes.* We examined the quality of bhandhobi relationships by inquiring about the amount of contact among bandhobi pairs. Of 12 pairs, six became friends, at least as evidenced by meetings 1–3 days a week. Three pairs communicated at least once fortnightly as required by the program. Three pairs did not contact each other outside meetings and may be viewed as having failed to form a close relationship.

## 4. Discussion

This pilot study sought to examine the feasibility and assess potential benefit of an innovative integrated depression treatment/economic strengthening intervention. The intervention showed robust impact on most measured outcomes, including depressive symptoms, tension, self-esteem, hope, social support, and participation in economic decision making. Although economic interventions can sometimes create a “spill-over” effect in economic interventions, in which control participants worsen on study outcomes, we saw little evidence of this effect, with control participants remaining mostly unchanged on study variables. The intervention also demonstrated excellent feasibility, with 100% of enrolled participants retained in the treatment and 100% of participants making their asset purchase at the end of 12 months.

Depression treatment is often beneficial, but, like other behavioral and psychological interventions, its potential to bring about lasting change has been increasingly called into question in recent years [66,67,68]. While depression treatment may provide a valuable benefit, it may have little impact on the patient’s social environment. Economic-strengthening interventions, by contrast, have the potential to change a woman’s status and position in her family and community. These impacts may have a lasting effect, as improving individual autonomy may result in changes in social relationships both within and outside the family. Depression treatment combined with economic strengthening may create therapeutic synergy, enhancing a patient’s autonomy and her potential to take advantage of the learning that occurs in depression treatment. The social support provided by the bandhobi component of the intervention may also have been beneficial.

The results reported here suggest that our integrated intervention impacted several dimensions of social and household empowerment, including participation in household financial decision making, and the reduction of experiences of physical and psychological coercion. Although our small sample size did not permit mediational analysis, the intervention also positively impacted other potential mediators such as hope, self-esteem, and social support.

Our study shows that depression treatment and economic strengthening can be combined successfully. Among the more notable findings are very high retention and attendance, with 100% participants receiving at least 10 sessions, and a median of 12 sessions. Retention in depression treatment studies is a major problem, with high dropout rates reported in many trials. In this study, the addition of an economic-strengthening component may have acted as a powerful incentive to remain in treatment. Another feature of the program, the “bandhobi” component, paired participants and encouraged regular engagement. Preliminary results suggest that at least half of the sample formed friendships with their partners. This may have helped to improve both wellbeing and treatment engagement.

Limitations of the study include its small sample size, which prevented an analysis of mediators of treatment impact. Furthermore, the sample size and study design did not permit the disaggregation of program components to determine which of several program features were the “active ingredients” of treatment. The short-term outcome (12 months) was another weakness: whether the integration of financial empowerment with depression treatment increases the persistence of treatment effects remains to be tested in a larger study. Additionally, apart from the achievement of the “match” and acquisition of a productive asset, this study did not have the capacity to fully assess economic outcomes. A final potential weakness was the size of the cash transfer, $186, which might have implications for long-term feasibility. Nevertheless, the size of the transfer is much smaller than those provided in recent poverty alleviation programs [53,54,69]. If our program is found to be effective in alleviating both depression and poverty over the long term, future studies could examine the impact of varying sizes of cash transfer.

## 5. Conclusions

This small-scale pilot study indicates both the feasibility and short-term effectiveness of an integrated depression treatment/economic empowerment intervention for low-income women in rural Bangladesh. The next steps are to test the model in a large-scale formal trial with a similar population. The larger study will examine whether this integrated intervention has the potential to make a long-term impact on the lives of participants. In addition to the impact on depression outcomes described here, future research should assess both economic outcomes and mediators/mechanisms of treatment impact. A complex, multi-armed clinical trial that compares different components—e.g., depression treatment alone, economic empowerment alone, and a mixed intervention—will help to answer this question.

## Figures and Tables

**Table 1:** Baseline demographics by group.

Variable	Intervention *n* = 24 Mean (SD)/%	Control *n* = 24 Mean (SD)/%	*p* Value
Age in years	26.0 (4.9)	26.1 (4.4)	0.98
Education (years in school)	6.0 (2.3)	4.4 (2.3)	0.03
Family size (number of people in household)	4.63 (1.5)	5.2 (1.91)	0.25
Family members (3 or more members)	45.9	54.1	0.16
Number of children (less than 2 children)	57.1	42.9	0.16
Monthly family income ($)	76.9 (17.7)	76.8 (22.2)	0.99
Loan/debt (US $)	35.2 (54.7)	40.0 (62.7)	0.78

Independent Sample *t*-test.

**Table 2:** Study outcomes.

Variable (Range)	Intervention (*n* = 24)			Control (*n* = 24)			*p* Value (95% CI)
	Baseline (T0) Mean (SD)	12 mo. Follow Up (T1) Mean (SD)	Mean Difference of Scores (T1–T0)	Baseline (T0) Mean (SD)	12-mo Follow Up (T1) Mean (SD)	Mean Difference of Scores (T1–T0)	
PHQ-9 (0–27)	14.5 (2.5)	5.5 (2.8)	−9.0	14.5 (3.6)	14.9 (2.8)	0.5	<0.001 (7.4, 11.6)
Tension Scale (25–100)	55.0 (6.7)	32.9 (4.9)	−22.1	55.4 (6.8)	50.0 (7.2)	−5.3	<0.001 (11.9, 21.7)
Rosenberg Self Esteem (0–32)	18.5 (5.2)	21.7 (4.4)	3.2	18.6 (5.8)	18.5 (3.9)	0.1	0.040 (−6.0, −0.2)
Trait Hope Scale (12–48)	36.5 (5.5)	39.6 (3.2)	3.1	36.4 (5.8)	36.6 (4.3)	−1.8	0.005 (−8.2, −1.6)
MOS Social Support (19–95)	68.6 (18.7)	92.1 (7.2)	23.5	63.4 (21.0)	74.7 (17.1)	11.3	0.024 (−22.6, −1.7)
Tangible Support	16.3 (4.4)	19.7 (1.1)	3.4	15.8 (5.2)	17.3 (4.8)	1.5	0.153 (−4.6, 0.7)
Affectionate Support	8.8 (3.8)	14.1 (1.9)	5.4	9.2(3.9)	10.7 (3.9)	1.5	0.001 (−6.2, −1.6)
Positive Social Interaction	10.1 (4.4)	14.2 (1.8)	4.1	11.0 (4.0)	12.0 (2.7)	1.0	0.015 (−5.6, −0.6)
Emotional Support	30.5 (9.8)	39.3 (3.3)	8.8	24.3 (11.9)	30.9 (10.3)	6.6	0.443 (−8.0, 3.5)
Additional Support	3.0 (1.4)	4.8 (0.6)	1.8	3.0 (1.4)	3.8 (1.0)	0.8	0.030 (−1.8,−0.1)
Economic Decision Making (0–6)	3.5 (2.1)	5.0 (1.3)	1.5	3.8 (2.2)	3.6 (1.9)	−0.1	0.011 (−2.8, −0.4)
Family Size Decision Making (0–2)	1.5 (0.8)	1.8 (0.4)	0.3	1.6 (0.7)	1.6 (0.7)	0.0	0.252 (−0.8, 0.2)
Freedom of Movement (0–5)	3.7 (2.2)	3.1 (2.5)	−0.5	3.1 (2.5)	2.5 (2.6)	−6.3	0.916 (−1.7, 1.5)
Coercive Control (0–2)	1.4 (0.6)	0.9 (0.9)	−0.5	1.3 (0.8)	1.4 (0.7)	0.1	0.011 (0.2, 1.2)
Gender Role Norms (0–5)	2.2 (0.4)	2.4 (0.8)	0.3	2.2 (0.5)	2.2 (0.7)	0.04	0.355 (−0.7, 0.2)

Unadjusted comparison (difference-in-difference analysis).

**Table 3:** Multivariable-adjusted linear regression analysis of the effects of intervention on social and psychological outcome measures at 12 months.

Outcome Measure	Regression Coefficient (Unstandardized) B ± SE (95% CI)	Standardized Coefficients Beta (β)	*p* Value	Effect Size
PHQ-9 score	−9.2 ± 0.8 (−10.9, −7.5)	−0.8	<0.001	−2.5
Rosenberg Self Esteem	3.5 ± 1.4 (0.7, 6.4)	0.3	0.017	0.60
Trait Hope	4.0 ± 1.1(1.8, 6.2)	0.5	0.001	0.68
Tension Scale	−15.1 ± 1.8 (−18.8, −11.4)	−0.7	<0.001	−2.2
MOS Social Support	16.7 ± 3.6 (9.4, 24.0)	0.5	<0.001	0.79

Intervention effect B is the regression coefficient. Ranges shown are 95% CIs. Results were adjusted for relevant baseline measures.

## Data Availability

Data is available on request via communication with the senior author.

## Abbreviations

Patient Health Questionnaire: PHQ; Human Immunodeficiency Virus: HIV; Global Mental Health: GMH; World Health Organization: WHO; Low- and Middle-Income Countries: LMIC; Matched Savings Accounts: MSA; National Development Programme: NDP; Medical Outcomes Study: MOS.
